# Cost of illness studies on reproductive, maternal, newborn, and child health: a systematic literature review

**DOI:** 10.1186/2191-1991-3-24

**Published:** 2013-11-11

**Authors:** Sanjib Saha, Ulf G Gerdtham

**Affiliations:** 1 Center for Primary Healthcare Research, Skåne University Hospital, Lund University/Region Skåne,, Jan Waldenströms gata 35, Malmö SE-205 02, Sweden; 2 Health Economics & Management, Institute of Economic Research, Lund University, Tycho Brahes väg 1, SE- 220 07, Lund, Sweden; 3 Department of Economics, Lund University, Tycho Brahes väg 1, Lund SE- 220 07, Sweden

**Keywords:** Cost of illness, Reproductive health, Maternal health, Breastfeeding, Preterm birth

## Abstract

**Background:**

The term “reproductive, maternal, newborn, and child health (RMNCH)” describes an integrated continuum of health states which is central to Millennium Development Goals 4 and 5. While the burden of mortality and morbidity associated with RMNCH is well known, knowledge is still limited about the economic burden of RMNCH. Concrete evidence of cost of illness (COI) of RMNCH may help policy makers in supporting investment in RMNCH.

**Methods:**

A systematic literature search of COI studies was performed in electronic databases. The time frame for the analysis was January 1990 – April 2011. The databases checked were Medline (Pubmed), Embase and ECONbase, EconLit, the Cumulative Index to Nursing and Allied Health (CINAHL), the National Bureau of Economic Research, the Latin American and Caribbean Literature on Health Sciences Database (LILACS), and Popline. Furthermore, we searched working papers and reference lists of selected articles.

**Results:**

All the studies investigated address particular complications and issues of RMNCH, e.g., preterm birth, non-exclusive breastfeeding, and sexually transmitted diseases (STDs), but not RMNCH as an entire continuum. Most of the studies were conducted in high income countries, with limited data on low and middle income countries. The burden of disease is very high even for single complications. For example, the disease burden related to non-exclusive breastfeeding was given as 14.39 billion international dollars (ID) (2012, purchasing power parity) per year in the USA. Methodological differences in study design, costing approach, perspective of analysis, and time frame make it difficult to compare different studies.

**Conclusion:**

The continuum of RMNCH covers a large portion of the lifespan from birth through the reproductive age. From a methodological perspective, an ideal COI study would clearly describe the perspective of analysis and, hence, the cost items (direct or indirect), cost collection procedure, discounting, quality of data, time frame of analysis, related comorbidities, and robust sensitivity analysis for all the assumptions. Further research is needed to measure the economic impact of RMNCH, including identification of the most cost-effective policy and interventions for prevention, reduction, and elimination of the complications of RMNCH.

## Review

### Background

“Reproductive, maternal, newborn, and child health (RMNCH)” refers to an effective and integrated continuum of care that delivers essential services and interventions to women facing particular risk arising from reproduction and pregnancy, their infants at critical points, and children in their first 5 years of age [1]. An RMNCH program is fundamental to development, as reflected in Millennium Development Goals (MDGs) 4 (reducing under-5 child mortality by two-thirds between 1990 and 2015) and 5 (reducing maternal mortality by three-quarters between 1990 and 2015 (5A), and achieving universal access to reproductive health by 2015 (5B) [2].

The current global under-5 mortality rate needs to be halved from 57 deaths per 1,000 live births to 29 to reach the target by 2015 [3]. Even when a country is on track with the goals, deep inequalities still persist, with the poorest communities continuing to experience far less progress than the richest [4]. Despite increased attention to maternal mortality and presence of interventions to address maternal health, current health systems and financial commitments for RMNCH may not be sufficient to achieve MDG 5A [5]. Research has shown that 80% of deaths in the maternal group could be averted if women had access to essential maternity and basic health care [5,6]. Reproductive health, including family planning, saves infant and maternal lives and reduces unintended births [7]. The progress towards MDG 5B is slow and uneven. In 2009, the contraceptive prevalence rate was 63% globally [8], but one in four Sub-Saharan African women still had an unmet need for family planning. Globally, during 1990–2009, 65% of births were attended by a skilled health worker, compared to 48% in Africa [9]. There has been insufficient investment to ensure that maternal and reproductive health services are accessible, affordable, and available on an equitable basis [10].

Cost of illness (COI) studies identify different components of costs of particular diseases or disease-related complications in different sectors of the society, which might have been saved if the diseases did not exist. This information can help policy makers to grasp the economic burden of the diseases or complications, and justify interventions [11]. Research of the economic burden of RMNCH diseases seems inadequate [12] and a concise review of available knowledge and evidence is required. Therefore, the aim of this report was to systematically review the literature on COI or disease burden for RMNCH and related complications.

## Methodology

### Methodological issues in cost of illness studies

Cost is the value of a resource, conceptually defined as the value that could be gained by using the resource in an alternative way. Economists use the concept “opportunity cost” or “economic cost” when conducting COI studies. Opportunity cost is the cost of an alternative that must be forgone in order to pursue an action or intervention. It is assumed that scarce resources always have a cost even if no money is spent because the resources could be efficiently used elsewhere.

The key idea behind economic cost estimation is that when resources are used to provide health care for one person, they are simply not available for other people or alternative societal uses. The methodological aspects of COI can be organized into the following broad categories:

#### Types of costs

The economic costs of a disease can be classified as direct, indirect, and intangible costs. Direct costs are all direct medical cost and direct non-medical costs. Direct medical costs are expenditures for diagnosis, treatment, continuing care, rehabilitation, and terminal care for an illness. Direct non-medical costs are the costs of non-health care resources, such as transportation to and from health care providers, certain household expenditures, costs of relocating and certain property losses, legal and court costs, and informal care [13]. Informal care is the care provided by family members or friends to the sick person.

Indirect cost is the productivity loss cost due to morbidity and mortality. There is a misperception of the term “indirect costs” in COI studies because it is used for costing of overhead and other shared costs at the patient level within the health care services accounting framework. Some researchers have suggested substituting the term “indirect costs” with “productivity costs” [14]. However, in this article, we use “indirect cost” in line with the previous World Health Organization (WHO) report [15].

The intangible costs capture the psychological dimensions of illness, including pain, bereavement, anxiety, and suffering. These types of costs are hard to measure and are usually not included in COI studies [11].

#### Perspectives

Cost of illness studies may be conducted from different perspectives which determine the types of cost included in the analysis. These perspectives may measure costs to society, the health care systems, participants and their families, and third party payers (Table 1).

**Table 1:** **Costs included in cost of illness (COI) studies using different perspectives**^**a**^

**Perspective**	**Medical cost**	**Cost of lost productivity (due to morbidity and mortality)**	**Non-medical cost (time cost, informal care, transportation)**	**Transfer payment**
Societal	All	All	All	Administration cost and excess burden of taxes
Health care system	All	–	–	–
Participants and their families	Out of pocket costs	Lost wages or household production	Out of pocket costs	Amount received
Third party payer	Covered cost	Covered cost	–	Amount paid by others + administration cost

Adapted from Gold et al. [14] and Segel JE [16]. ^a^This is a general categorization and may not apply to all cases.

The perspective of COI studies needs to be clearly stated because each study included covers slightly different costs. The purpose of a study ultimately determines the perspective. A study that takes the health care systems perspective needs to include only medical care-related costs. The societal perspective is more comprehensive and is consequently often recommended by researchers [14]. The societal perspective includes all costs (direct and indirect) except transfer payments (a shift of resources such as social security benefits or Medicare, Medicaid payments) [16].

#### Costing approach

Cost of illness studies may be based on different combinations of costing approaches, as discussed below, in their application:

• Incidence or prevalence-based approaches

• Top-down or bottom-up approaches

• Prospective or retrospective approaches

##### Incidence and prevalence-based studies

Incidence-based studies estimate the lifetime costs of a disease from its onset to its termination, which include the discounted morbidity and mortality costs for the incident cohort, usually calculated based on the year when the disease first appeared. Morbidity costs are defined as the value of income lost from decreased productivity, restricted activity, absenteeism, and bed days. Mortality costs are the value of future income lost by premature death.

Prevalence-based studies estimate the costs of all disease cases (new as well as pre-existing) in a given year. They include all medical care costs and morbidity costs for a disease within the study year.

Each approach has benefits and drawbacks. However, prevalence-based studies are rather common because they require less data and fewer assumptions compared to incidence-based studies, and are less expensive to conduct. The prevalence-based approach is suitable for measuring the total current economic burden of a disease. Prevalence-based studies provide a snap shot of the disease and may not capture the progression of the disease at various stages. Therefore, they may capture the end stage of a disease which may not be avoidable. For this reason, prevalence-based studies are not perfectly suitable for measuring the potential savings from preventive interventions. They are, however, suitable for diseases where costs remain relatively stable over a time period. Incidence-based studies provide an estimate of the savings potentially accrued if the preventive measure is implemented. These are also helpful in analyzing the management of illness from the onset of disease till recovery or death. This provides a better picture of how the cost increases or decreases as the diseases progresses and consequently allows policy makers to plan interventions targeting specific stages of disease and/or specific population groups [11,13,16].

##### A top-down (population-based) or bottom-up (person-based) approach

Two approaches that are commonly used for quantifying the resources are the top-down (population-based) and the bottom-up (person-based) approach. The top-down approach estimates economic costs by using aggregate data on mortality, morbidity, hospital admissions, general practice consultations, disease-related costs, and other health-related indicators. Various sources and types of data are used to calculate the fractions of resources used that can be attributed to each disease. Generally this information is collected from national health care statistics, patient registers, insurance databases, etc. One disadvantage of the top-down approach is that not all costs are usually included in the database (e.g., costs for informal care and the patient’s time cost are not included). For complex diseases, the top-down approach may underestimate or overestimate the costs caused by comorbidities related to the disease of interest and there is a risk of misclassification if a diagnosis-based classification is used. The top-down approach is limited to providing cost estimates stratified by disease subtypes, severity, and patient characteristics and demographic variability. The bottom-up approach calculates the resources used and productivity loss in individuals with the health problem in question. The mean per-person costs are then extrapolated to the whole population with relevant epidemiological data. In this case, the patient sample size needs to be unbiased and representative of the national population. The bottom-up approach is more comprehensive and enables detection of the variability related to differences in important demographic characteristics between patients [11,13,16].

##### Prospective or retrospective cost of illness studies

In retrospective COI studies, all events have already occurred by the time the study is initiated. The researchers go back to collect the resources used and adjust these to the base year price. By contrast, in prospective COI studies, the relevant events have not yet occurred when the study is initiated and the researchers follow patients over time to collect the data. Both prevalence and incidence studies can be either prospective or retrospective. Retrospective studies are less expensive and less time-consuming; however, they can only be carried out when sufficient data is available. In prospective analysis, researchers can design the data collection methods and get details of cost items (e.g., patient transportation cost, time cost), which may not be possible in retrospective analysis without any additional assumptions. Prospective analysis is not suitable if the duration of disease is long, which might affect the participant and therefore hamper the quality of the data [11].

#### Discounting

Discounting is a method used to capture an individual’s preference for income today rather than his or her income in the future. Discounting allows calculation of the present value of payments that will occur in the future. The appropriate discount rate varies in the scientific literature but the WHO uses a 3% discount rate [17].

#### Indirect cost calculation method

Although there is not universal agreement about including indirect cost in COI, or about how to estimate indirect costs, indirect cost calculation is common practice. There are two commonly used methods: the human capital approach (HCA) and the friction cost approach (FCA). The HCA estimates total production losses due to illness, premature death, or disability by calculating the total period of absence and multiplying this by the average wage rate of the absent worker. Calculations using HCA often include the value of household work, usually valued as the opportunity cost of hiring a replacement from the labor market. The FCA only estimates the actual production that is lost during the time it takes to replace the ill worker. Whereas the HCA reflects lost productive potential, the FCA measures actual production losses. Both methods have pros and cons, which has been elaborately discussed elsewhere [15].

#### Sensitivity analysis

Researchers perform sensitivity analysis for measuring the degree of uncertainty. In sensitivity analysis, the key variables (unit price, incidence or prevalence rate of the diseases, discount rates, etc.) are changed to assess the robustness of the result. Sensitivity analyses can be one-way, i.e., changing the parameter of one variable, or multi-way, i.e., changing the parameter of two or more variables. Probabilistic sensitivity analysis includes the variables as a distribution and provides the probabilistic behavior of a model [17].

## Literature search

We performed systematic literature searches in electronic databases such as Medline (Pubmed), Embase, ECONbase, EconLit, the Cumulative Index to Nursing and Allied Health (CINAHL), The National Bureau of Economic Research, the Latin American and Caribbean Literature on Health Sciences Database (LILACS), and Popline. In addition, we also searched the homepages of some major international organizations such as the World Bank, the WHO, Save the Children (UK & USA), the UK Department for International Development (DFID), the United Nations Children’s Fund (UNICEF), the United Nations Population Fund (UNFPA), and Guttmacher Institute. We also performed searches from the reference lists of included studies and reviews. The search strategy including keywords is presented in Annex 1 (Additional file 1).

The article search was limited to the period January 1990 – April 2011. We selected studies in the English language related to RMNCH and focusing economic burden. Exclusion criteria were economic evaluation studies related to RMNCH, such as cost minimization, cost effectiveness, cost utility, and cost benefit analysis. We also excluded notes, commentaries, and editorials related to RMNCH and published in scientific journals.

**Figure 1:**
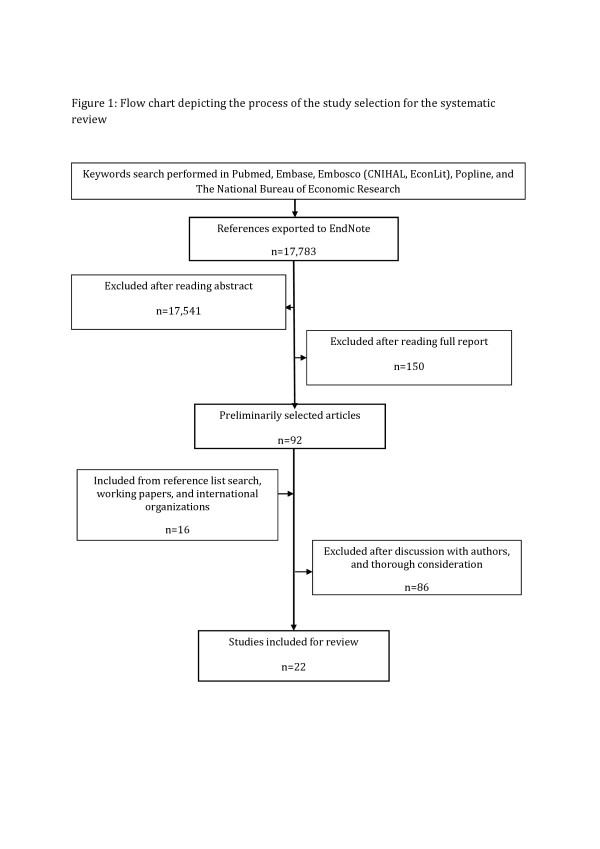
Flow chart depicting the process of the study selection for the systematic review.

The initial hits from the electronic databases were exported to EndNote and checked for duplication. Thereafter, we screened the articles by reading the abstracts. We further searched for articles and reports on websites of international organizations. We searched reference lists of preliminarily selected articles and discussed disagreement about any selection. Figure 1 provides a flow chart of the article selection process.

## Results

The purpose of this review is not to discuss the strengths and weaknesses of each individual article, but rather, to give a general picture of the literature and discuss overall methodological issues which might potentially influence results, such as costing methods, cost items, discount rate, etc.

The continuum of RMNCH covers a large portion of the lifespan, from birth through reproductive age. We found that all the studies addressed particular complications or areas of RMNCH, e.g., non-exclusive breastfeeding, preterm birth, tuberculosis, reproductive health, but not RMNCH as an entire continuum. We have grouped the studies into RMNCH-related complications (Table 2). For better comparison, we have also converted costs to international dollars (ID) based on purchasing power parity (PPP) (2012).

**Table 2:** Characteristics of the studies included

**Study, publication year, country**	**Costing year**	**Topic related to RMNCH**	**Perspective**	**Time frame**	**Cost**		**Costing approach**			**Cost items and description**		**Discount rate**	**Sample size**
					**As reported**	Purchasing power parity (PPP) in 2012, given as international dollar (ID)*	**Incidence/prevalence**	**Bottom- up/top-down**	**Retrospective/prospective**	**Direct cost**	**Indirect cost**		
Bartick & Reinhold, 2010, USA [18]	2007	Breastfeeding	Societal	1 year	US$13 billion/year with 90% compliance rate and US$10.5 billion/year with 80% compliance rate	ID14.39 billion/year with 90% compliance and ID11.62 billion/year with 80% compliance	Not clear	Not clear	Not clear	Disease-specific costs are obtained from the literature	Not clear	3%	Not clear
Buchner et al., 2007, the Netherlands [21]	Not clear	Breastfeeding	Health care	1 year	€50 million/year for 6 months’ exclusive breastfeeding	ID47 million/year for 6 months’ exclusive breastfeeding	Incidence	Not clear	Not clear	Disease-related cost from the Netherlands	NA	4%	Model simulation
Ringborg et al., 2006, Sweden [23]	2000	Preterm birth	Not clear	1 year	For LBW babies, mean cost €21,837/baby; for preterm births, €20,263/baby	For LBW babies, mean cost ID37,838/baby; for preterm births, ID35,119/baby	Not clear	Not clear	Retrospective	Only inpatient care cost	NA	NA	336,136 live births
Schmitt et al., 2006, USA [24]	2003	Preterm birth	Not clear	Hospital discharge	US$33,970/LBW child	ID42,308/LBW child	Not clear	Not clear	Not clear	Hospital care cost	Not clear	NA	Cohort of 518,704 live births
Petrou, 2003, UK [25]	1998–1999	Preterm birth	Not clear	10 years	£18,000 for babies of GA <31 weeks and £5,376 for babies of <37 weeks’ GA	ID18,604 for babies of GA <31 weeks and ID5,556 for babies of <37 weeks’ GA	Not clear	Not clear	Not clear	Hospital admission care	Not clear	Not clear	Cohort of 117,212 births
Phibbs & Schmitt, 2006, USA [26]	2003	Preterm birth	Not clear	Not clear	Delaying delivery from 26 to 37 weeks will save US$206,000/case, and from 29 to 37 weeks will save US$122,000/case	Delaying delivery from 26 weeks to 37 weeks will save ID25,7045/case, and from 29 to 37 weeks will save ID152,230/case	Not clear	Not clear	Not clear	Inpatient care cost	Not clear	Not clear	193,167 infants at 24–37 weeks’ GA
Mangham et al., 2009, UK [27]	2006	Preterm birth	Health care	18 years	£2,946 billion	ID2.10 billion	Incidence	Bottom-up	Retrospective	Inpatient and outpatient care	NA	3.5%	Hypothetical cohort of 669,601 children
Behrman & Bulter, 2007, USA [28]	2005	Preterm birth	Societal	Lifelong	US$26.2 billion	ID31.15 billion	Incidence	Top-down	Retrospective	Child cost and mother cost	Household and labor market productivity US$5.7 billion	3%	Cohort of 23,631 births
John et al., 2009, India [30]	2004	Tuberculosis	Societal	2004	US$311 million	ID35 million	Prevalence	Not clear	Not clear	Inpatient and outpatient care, medicine, diagnostics, medical appliances	Informal care cost, lost productivity cost	–	73,868 households
Rein DB 2000, USA [31]	1998	PID	Health care	1 year; lifetime	US$1.88 billion for 1 year and US$1,167/case for a lifetime	ID2.64 billion for 1 year; ID1,643 for a lifetime	Prevalence	Bottom-up	Retrospective	Inpatient, outpatient, and STD clinic cost	NA	5%	1.76 million visits to clinic
Yeh at al., 2003, USA [32]	2000	PID	Societal	Lifetime	US$1,060–3,180/person over a lifetime	ID1,413–4,239/person over a lifetime	Not clear	Not clear	Not clear	Only direct medical costs derived from the literature	Lost productivity cost	3%	Hypothetical cohort of 100,000
Trent et al., 2010, USA [33]	2009	PID	Health care	1 year	US$3,025/episode	ID3,237/episode	Prevalence	Bottom-up	Retrospective	Inpatient and outpatient costs	NA	Not clear	152 individuals
Owusu-Edusei et al., 2010, USA [34]	2007	Chlamydia	Third party	Per episode	US$141 for females/episode and US$157 for males/episode	US$156 for females/episode and US$173 for males/episode	Prevalence	Not clear	Retrospective	Hospital care cost	NA	Not clear	7,301 male, 26,313 female cases
Blandford & Gift, 2006, USA [35]	2001	Reproductive health	Not clear	Lifetime	US$130 per chlamydia infection and US$649 per PID	ID168 per chlamydia infection and ID841 per PID	Not clear	Not clear	Not clear	NA	Productivity lost, days (HCA)	3%	Monte Carlo simulation
Pultorak et al., 2009, USA [36]	2007	STI	Health care	2 years	US$69.7 million for chlamydia, gonorrhea, and syphilis	ID77.18 million for chlamydia, gonorrhea, and syphilis	Incidence	Not clear	Not clear	From the literature	NA	No discount	Not clear
Chesson et al., 2004, USA [37]	2000	STDs	Health care	1 year	US$6.5 billion in 2006	ID8.66 billion	Incidence	Bottom-up	Not clear	From the literature	NA	3%	Not clear
Hoy et al., 2009, USA [39]	2004	Genital warts	Third party payer	1 year	US$220 million	ID267.39 million	Prevalence	Bottom-up	Retrospective	Diagnosis, treatment, outpatient visits	Not clear	NA	1,158 patients from a cohort & US census
Hillemanns et al., 2008, Germany [40]	2005	Genital warts	Third party and societal	1 year	€49.0 million third party cost; €54.1 million societal cost	ID49.53 million third party cost; ID54.68 million societal cost	Prevalence	Bottom-up	Retrospective	Outpatient visits, diagnostic test, hospitalization, medication	Loss of productive days, calculated as GDP/person/day	NA	Statistically extrapolated for the entire German population
Insinga et al., 2003, USA [41]	2000	Genital warts	Third party perspective	1 year	US$140 million	ID186.66 million	Prevalence	Bottom-up	Retrospective	Outpatient, inpatient, and pharmaceutical care	Not clear	NA	1,919 patients and extrapolation
Pirotta et al., 2010, Australia [42]	2009	Genital warts	Health care	1 year	AUS$14 million	ID21.52 million	Prevalence	Bottom-up	Retrospective	GP visit, GP referral, and hospital care	–	NA	Extrapolation to the whole country
Marra et al., 2009, Canada [43]	2006	Genital warts	Health care	8 years	Can$8,295,101, or Can$1 million per year	ID11,402,469, or ID1.34 million per year	Not clear	Not clear	Retrospective	Inpatient care, physician time, nursing, drugs	NA	No discount	39,493 incident cases and 50,634 prevalent cases

*Data obtained from the Organization for Economic Cooperation and Development (OECD) (http://stats.oecd.org/Index.aspx?DatasetCode=SNA_TABLE4), United States Department of Labor, Bureau of Labor Statistics (ftp://ftp.bls.gov/pub/special.requests/cpi/cpiai.txt), and The World Bank (http://data.worldbank.org/indicator/SL.GDP.PCAP.EM.KD?page=1).

GA= gestational age; HCA = human capital approach; NA = not applicable; PID = pelvic inflammatory disease; RMNCH = reproductive, maternal, newborn, and child health; STD = sexually transmitted disease; STI = sexually transmitted infection.

### Breastfeeding

Two of the included studies estimate the cost for non-optimal breastfeeding duration, one in the USA and the other in the Netherlands. The US study [18] is an updated version of a previous article [19], and considers diseases which might be benefited by breastfeeding, such as lower respiratory tract infection, atopic dermatitis, sudden infant death syndrome, childhood leukemia, childhood asthma, type 1 diabetes mellitus, and obesity. It reports that if 90% of US families breastfed their child for 6 months (current rate: 12.5%), this would save US$13 billion (2007 price year) and prevent 911 deaths per year; if 80% did, this would save US$10.5 billion and prevent 741 deaths per year. Loss of earnings from premature death was a major contributor to this loss, comprising US$9.5 billion (73% of 13 billion), and may be overestimated because the “revealed preference job risk” approach is not totally unbiased [20]. However, the researchers considered the benefits to the children only, while breastfeeding also has potential health benefits for mothers. Consequently, the calculated cost may not give a true picture.

By contrast, the Dutch study [21] considered benefits of breastfeeding for both children and mothers. The authors used the Netherlands Institute for Public Health and the Environment (RIVM) chronic disease model [22] to estimate national savings related to eight diseases/disorders for children (otitis media, gastrointestinal infection, asthma, respiratory infection, eczema, Crohn’s disease, leukemia, and obesity) and three diseases for mothers (rheumatic arthritis, premenopausal breast cancer, and ovarian cancer). The total annual cost saving from 6 months’ exclusive breastfeeding was estimated to be EUR50 million. The disease-specific costs were based on data specific to the Netherlands.

### Preterm birth

Two studies [23,24] have estimated inpatient costs for preterm births, one in Sweden and the other in the USA. The costs were almost three times higher for preterm (<37 weeks’ gestation) and low birth weight (LBW) (<2,500 g) compared to normal weight, full term babies. In the US study (24), costs are given as hospital-specific cost-to-charge ratios, whereas in the Swedish study (23), calculation of costs was based on Diagnostic Related Group (DRG) criteria. The US study differentiates between costs for infants and costs for mothers and includes costs up to the baby’s discharge or death [24]. In the USA, the median cost for very low birth weight (VLBW), LBW, and normal weight babies is given as US$93,481, US$7,141, and US$570, respectively (2003 prices). By comparison, in Sweden, the cost for a preterm baby is €20,263 (2000 prices). About 60% of mothers constituted 60% of the total maternal cost, whereas 5% of LBW and VLBW children accounted for 75% of the child cost. The time frame for these studies was 1 year. A UK-based study estimating cost for the first 10 years of life [25] reports that the long term inpatient care cost is 443% higher in preterm compared to term babies. All three studies conclude that cost decreases as the birth weight increases and/or with term/near-term delivery [23-25].

One study investigated cost savings with delay of one preterm delivery by ≥1 week [26]. The authors show that with each week’s delay in delivery, the cost decreases. Delaying deliveries at 29 weeks to 37 weeks would save US$122,000 per case (2003 price year). The saving would be even greater for deliveries at <26 weeks: US$206,000 per case. These estimations only consider inpatient costs. However, LBW babies suffer from various comorbidities in childhood and adulthood. Mangham et al. [27] calculated the cost of preterm birth in England and Wales up to 18 years: to this purpose, they developed the Markov model to analyze a hypothetical cohort of children to represent the total number of live births in England and Wales. These costs include hospital inpatient and outpatient care, community health care, social care, and special education cost. They considered only four types of disability with different levels of severity: motor (including cerebral palsy), visual, and hearing impairment, and development delay or cognitive disability. They gave the total cost of preterm births in England and Wales as US$4,567 billion (2006 price year) [27]. This may, however, be an underestimation as only four types of disability for children but no costs related to maternal illness were considered.

One study [28] analyzed the lifelong cost of preterm birth for mothers and children from a societal perspective. Disabilities for preterm children considered were the same as mentioned previously. Maternal care costs included the costs of prenatal care, delivery services, costs associated with pregnancy morbidity, and precautionary care. Caregiver costs included travel costs and time costs for caring for a preterm baby. However, the study did not explicitly state whether the caregiver was formal or informal. The total societal burden of preterm birth was given as US$26.2 billion (2005 price year). The indirect cost, US$5.7 billion, was for loss of household and labor productivity [28]. The authors did not consider productivity cost for premature death as they assumed that all the infant deaths would be replaced and there would be no productivity cost from premature death. Although the collected cost data were for the whole country, the data given represent only one state.

### Tuberculosis

One study [29] estimating the global burden of tuberculosis (TB) included only calculation of the productivity lost due to TB-related morbidity and mortality. The morbidity-related cost comprised a 30% reduction in average productivity for 8.4 million sick people worldwide, i.e., US$1 billion. Two million annual deaths cost an average loss of income of US$11 billion, giving US$12 billion annually for the TB burden. However, the study does not detail the methodology of estimation.

John et al. [30] estimated the cost of tobacco consumption in India, one of the complications being tuberculosis. Using the prevalence-based attributable risk approach, they estimated the relative risk (RR) of tobacco mortality in a cohort of 99,570 people. Their data on inpatient and outpatient cost of tobacco-related morbidity was from a national sample survey. In 2004, TB accounted for a cost of US$311 million in India. However, using RR as attributable risk may be conservative although the method is widely used in the literature. Moreover, the cohort represents people aged ≥35 and may therefore not capture the true effect of smoking. Furthermore, the cost of premature death has not been included in the analysis due to lack of data. The results may therefore be an underestimation.

### Reproductive health

Rein [31] measured the total economic burden of pelvic inflammatory diseases (PIDs) in the USA from a health care perspective. They analyzed 3 year PID claims data to collect the unit cost, and used a probability model to estimate the total disease burden and lifetime costs. The lifetime cost was US$1,167/case at 1998 prices. Using a Markov model, Yeh et al. [32] estimated the lifetime costs for PID, from a societal perspective, in the USA. Their cost calculation may be an overestimate as four of the studies they collected data from were based on some assumption which was not taken care of (e.g., not nationally representative, dated inpatient cost). The lifetime cost was US$1,060–3,180 per person at 2000 prices. Trent et al. [33] likewise calculated the direct cost of PIDs, but for adolescents only. They collected cost and claim rates from physicians and hospitals, a total of US$3,025 per episode, for 1 year in 2009. Owusu-Edusei et al. [34] estimated the cost of chlamydial infection in the USA for 2003–2007 from a third party payer (employer and private insurance) perspective. They collected disease rates in 7,301 males and 26,313 females from insurance databases. The average cost was US$157 for males and US$141 for females. Considering the total annual incidence of around 2.8 million, this cost is enormous from an employer perspective. Blandford and Gift [35] estimated the lifetime productivity cost attributable to *Chlamydia trachomatis* using the Monte Carlo model with the HCA. The study was limited to women aged 15–44. The mean weighted productivity loss was approximately US$130 dollar per untreated chlamydia infection and US$649 per PID (2001 prices).

Pultorak et al. [36] calculated direct medical costs of chlamydia, gonorrhea, and syphilis in the USA. They concluded that the incidence is higher among 20–24-year-olds and blacks. The cost burden was US$69.7 million for 2 years at 2007 prices. However, they may have underestimated costs because many STD cases are not reported and remain undiagnosed.

Chesson et al. [37] estimated the costs of sexually transmitted diseases (STDs) in 15–24-year-olds in the USA. They considered direct costs for eight major diseases: HIV/AIDS, human papillomavirus (HPV), genital herpes simplex virus type 2, hepatitis 2, hepatitis B, chlamydia, gonorrhea, trichomoniasis, and syphilis. The total burden was estimated to be US$6.5 billion (2000 price year). In an earlier report [38], the disease burden for the same eight diseases in the USA was estimated at US$8.6 billion (1997 prices).

Cost of illness studies on genital warts are mainly from developed countries and give third party payer perspectives [39-43]. In most cases, prevalence and incidence rates are based on epidemiological studies and extrapolated for the whole country. All the articles provide the age- and gender-specific distribution of diseases. All show that the peak occurrence of the disease happens later in life in men than in women. The cost estimations are based on patient registry databases throughout. None of the studies used prospective cost analysis. Insanga et al. [41] included inpatient care costs, whereas others did not. The duration in all included studies on genital warts was 1 year except for a study from Canada [43], which spanned 8 years.

## Discussion

All the studies reviewed considered particular complications related to RMNCH, which all cost substantial resources. For example, non-exclusive breastfeeding cost ID14.39 billion in the USA compared to ID56 million in the Netherlands. Preterm birth cost ID2.96 billion and ID30.80 billion in the USA. Genital warts cost ID186 million in the USA, ID23 million in Australia, and ID1.35 million in Canada.

Different approaches used for estimating direct and indirect costs have, however, limited the comparability of studies. Important methodological limitations are: reliance on administrative datasets without checking data quality; failure to clarify the perspective of analysis, and omission of key cost items; small sample sizes and selection bias; lack of sensitivity analysis; use of charges or inadequately described costing methods; lack of controls; inadequate follow-up; and failure to include discounting in longer-term studies. Most studies do not disaggregate costs into their components, making it difficult to target the source and predictors of high costs. All the cost estimations ignore at least some potentially important components of costs and are therefore likely to underestimate true resource use. These cost components include professional fees, transportation costs, maternal pregnancy complications, postpartum complications and out of pocket costs for parents, and informal care costs. There is almost no information on the lost earnings of parents and other family members for infant care or on productivity losses regarding the infants themselves.

One key issue is the inclusion of comorbidities for particular health problems. Improved study designs and long term cohorts are providing important epidemiological findings, but agreement still needs to be reached in the scientific community about the diseases benefited by particular interventions. For example, the US study [18] has included type 1 diabetes in its estimation, whereas the Dutch study [21] does not include breastfeeding in its COI calculations. Type 1 diabetes has significant cost burdens throughout life [44]. The same issue has been examined in preterm birth studies [25-28], where LBW was reported to affect many diseases through infancy, childhood, and even adulthood. The cost and benefits for both infant and mother are interlinked and most of the studies have failed to capture this. Regarding breastfeeding, the US study [18] does not include any benefit to the mothers, whereas the Dutch study [21] includes three diseases that may be benefited by exclusive breastfeeding, with substantial cost reduction. Maternal costs for delivery and hospitalization and the costs of antenatal admission are excluded in most studies. There also is a risk of double counting when calculating both maternal and child cost together. The analysis of cost for infants and mothers can be done separately, with possible risk of underestimation of the costs of prematurity and the possibility of missed shifting of costs between the two groups. Regarding preterm births, few studies have addressed educational costs, and those that have, do not provide adequate information across the spectrum of school age and disability.

Many of the studies are based on disease-specific costs collected from the literature [18,21,26-28,37]. This is a common practice in COI research; there needs to be some adjustment in, as well as agreement on, the transferability of different cost studies. In most of the articles, it is not stated whether they were the result of systematic reviews. Also, the methodology for costing and cost adjustment is not given, e.g., prevalence-based vs. incidence-based, or a bottom-up vs. a top-down approach [16,45].

The HCA is the most popular method for estimating loss of productivity cost in societal perspective studies. Using an average wage rate for the total population has the advantage of encouraging social equity in the distribution of health services and other resources, because lost productivity resulting from morbidity and mortality is valued equally across persons. However, this method produces biased estimation because for some diseases, the incidence is not distributed uniformly across populations. A better strategy may be to take account of the age distribution specific to the disease and the corresponding wage rates specific to the affected age. None of the studies have tried to capture the intangible cost of STDs in terms of human suffering, pain, grief, and social stigma. Moreover, the harmful impact of STDs on infants leads to long term emotional suffering and stress for families, which is almost impossible to capture in monetary terms. But this cost may theoretically be included in the estimation.

We also found disagreement regarding the indirect cost of infant death. For example, in one study [18], the cost of premature death of infants for any type of disease was US$10.5 million; in another study [28], this cost was not considered. The author argued that premature infants’ death and the loss of their productivity could be replaced.

Most of the studies obtained data on a particular cohort or region and extrapolated this to the entire country. Extrapolation of health care utilization and costs across geographical areas is potentially confounded by differences in demographics, including the underlying health status of the populations, as well as by differences in health care delivery conventions by provider. For this reason, adjustment of charges to costs and adjustments of costs for differences across geographic areas may not be sufficient in projecting cost estimates from one region to the nation as a whole. Risk adjustment based on differences in population characteristics and further adjustment for organizational differences in care delivery may be required.

Some studies use decision analytic models to estimate cost, e.g., the Markov model [27,32] and Monte Carlo simulation [35]. Inclusion of models requires a clear description of the assumptions and proper sensitivity analysis to verify the uncertainty.

Some researchers argue that COI estimates largely quantify transfers paid from one entity to another and are not indicative of actual costs [13]. Moreover, intangible costs, such as pain, suffering, grief, and social stigma, caused by illness are largely ignored in COI studies. Consequently, COI estimates may either substantially overestimate or underestimate the true financial burden of diseases. Furthermore, even if COI estimates correctly quantify all costs, critics still question the value of the estimates [11]. By focusing on health sector spending and lost labor productivity only, COI studies provide only a partial picture of the true macroeconomic impact of disease. They fail to consider ways in which depleted capital accumulation, investment in human capital, and demographic change contribute to diminished economic growth.

One prominent function of COI calculations is setting priorities in decision making. What is important is not the size of burden of a disease per se, but rather, how it can be reduced by preventive and/or therapeutic interventions [46]. The rational use of resources to ensure maximum benefits can only be ensured by economic evaluation studies that account for costs of prevention and treatment, as well as outcomes of the interventions. Yet, reviews of economic evaluation focusing preventive and therapeutic interventions of RMNCH are limited. Therefore, the next step would be to systematically gather evidence concerning RMNCH interventions which have proven to be the best value for money.

One limitation of this study is that we only examined English language articles; also, there may be a lack of items weighting (Table 2). However, this is a first attempt to discuss the cost burden of interlinked RMNCH issues under one umbrella.

## Conclusion

This study could serve as the basis for projecting disease expenses and help international organizations like the Partnership for Maternal, Newborn, and Child Health (PMNCH) to pursue authorities to prevent diseases of/manage RMNCH and related complications and thus save societal resources.

## Competing interests

The authors declare that they have no competing interests.

## Authors’ contributions

SS and UGG were involved in all stages of literature search, review of studies, and revising manuscript. First draft of the manuscript was written by SS. Both authors read and approved the final manuscript.

## Supplementary Material

Additional file 1 Annex 1: Searching methods including keywords. Click here for file
