# Supplementary material for: Cost of illness studies on reproductive, maternal, newborn, and child health: a systematic literature review
Source: Health Econ Rev. 2013 Nov 11;3:24. doi: 10.1186/2191-1991-3-24 (PMC4177189; doi:10.1186/2191-1991-3-24)
Supplement: Additional file 1 — Annex 1: Searching methods including keywords. [file 2191-1991-3-24-S1.doc]

# Annex 1: Searching methods including keywords

**PubMed search**

Economic terms

1. "economics"[Mesh] AND "economics" [Subheading] OR "economics"
2. "economic development"[MeSH Terms] OR "economic development"[All Fields]
3. "economic impact"[All Fields]
4. "economic consequences"[All Fields]
5. "economic analysis"[All Fields] AND ("analysis"[Subheading] OR "analysis"[All Fields])
6. "investments"[MeSH Terms] OR "investments"[All Fields] OR "investment"[All Fields]
7. "cost of illness"[MeSH Terms] OR "cost of illness"[All Fields]
8. "disease burden"[All Fields]
9. "cost minimization"[All Fields]
10. "costs and cost analysis"[MeSH Terms] OR "cost analysis"[All Fields]
11. "efficiency"[MeSH Terms] OR "efficiency"[All Fields] OR "productivity"[All Fields]
12. "salaries and fringe benefits"[MeSH Terms] OR "salaries"[All Fields] OR "wages"[All Fields]
13. "financing"[All Fields]
14. "economic benefit"[All Fields]
15. "economic burden"[All Fields]
16. #1 OR #2 OR #3 OR #4 OR #5 OR #6 OR #7 OR #8 OR #9 OR #10 OR #11 OR #12 OR #13 OR #14 OR #15
17. #16 AND ("humans"[MeSH Terms] AND English[lang] AND ("1990/01/01"[PDAT] : "2011/04/15"[PDAT]))

Maternal terms

1. "mothers"[MeSH Terms] OR "mothers"[All Fields] OR "maternal"[All Fields]
2. maternity[All Fields]
3. pregnan*
4. "postpartum period"[MeSH Terms] OR ("postpartum"[All Fields]
5. "postpartal"[All Fields]
6. ("parturition"[MeSH Terms] OR "parturition"[All Fields] OR "childbirth")
7. "obstetrics"[MeSH Terms] OR "obstetrics"[All Fields]
8. "obstetric care"[All Fields]
9. "breast feeding"[MeSH Terms] OR "breast feeding"[All Fields] OR "breastfeeding"[All Fields]
10. "lactation"[MeSH Terms] OR "lactation"[All Fields]
11. #18 OR #19 OR #20 OR #21 OR #22 OR #23 OR #24 OR #25 OR #26 OR #27
12. #28 AND ("humans"[MeSH Terms] AND English[lang] AND ("2004/07/01"[PDAT] : "2011/04/15"[PDAT]))

Child terms

1. Child*
2. "infant, newborn"[MeSH Terms]OR "newborn"[All Fields]
3. "baby"[All Fields] OR "infant"[MeSH Terms] OR "infant"[All Fields]
4. "school age"[All Fields] OR "school-age"[All Fields]
5. "birth weight"[MeSH Terms] OR "birth weight"[All Fields]
6. "infant, low birth weight"[MeSH Terms] OR "low birth weight"[All Fields]
7. "premature birth"[MeSH Terms] OR "preterm birth"[All Fields]
8. "body height"[MeSH Terms] OR "body height"[All Fields]
9. Height [All Fields]
10. #30 OR #31 OR #32 OR #33 OR#35 OR #36 OR #37 OR #38
11. #39 AND ("humans"[MeSH Terms] AND English[lang] AND ("2004/07/01"[PDAT] : "2011/04/15"[PDAT]))

Reproductive health

1. Reproduct*
2. Contracep*
3. ("contraceptive devices"[MeSH Terms] OR "contraceptive devices"[All Fields])
4. ("contraceptive agents"[MeSH Terms] OR "contraceptive agents"[All Fields] OR "contraceptives"[All Fields] OR "contraceptive agents"[Pharmacological Action])
5. "family planning services"[MeSH Terms] OR "family planning"[All Fields]
6. "family planning policy"[MeSH Terms] OR "family planning policy"[All Fields]
7. "population control"[MeSH Terms] OR "population control"[All Fields]
8. "birth control"[All Fields]
9. abstinence[All Fields]
10. "abortion, induced"[MeSH Terms] OR "induced abortion"[All Fields] OR "abortion"[All Fields]
11. "abortion, spontaneous"[MeSH Terms] OR "spontaneous abortion"[All Fields] OR "miscarriage"[All Fields]
12. "stillbirth"[MeSH Terms] OR "stillbirth"[All Fields]
13. caesarean[All Fields]
14. "sexually transmitted diseases"[MeSH Terms] OR "sexually transmitted diseases"[All Fields]
15. "stds"[All Fields]
16. "chlamydia"[MeSH Terms] OR "chlamydia"[All Fields]
17. "herpes genitalis"[MeSH Terms] OR "genital herpes"[All Fields]
18. "hepatitis"[MeSH Terms] OR "hepatitis"[All Fields]
19. "gonorrhoea"[All Fields] OR "gonorrhea"[MeSH Terms] OR "gonorrhea"[All Fields]
20. "syphilis"[MeSH Terms] OR "syphilis"[All Fields]
21. "genital warts"[All Fields]
22. "hiv"[MeSH Terms] OR "hiv"[All Fields]
23. "acquired immunodeficiency syndrome"[MeSH Terms] OR "acquired immunodeficiency syndrome"[All Fields] OR "aids"[All Fields]
24. "sexually transmitted diseases"[MeSH Terms] OR "sexually transmitted infections"[All Fields]
25. STIs[All Fields]
26. #41 OR #42 OR #43 OR #44 OR #45 OR #46 OR #47 #OR #48 OR #49 OR #50 RO #51 OR #52 OR #53 OR #54 OR #55 OR #56 OR #57 OR #58 RO #59 OR #60 OR #61 OR #62 OR #63
27. #66 AND ("humans"[MeSH Terms] AND English[lang] AND ("1990/01/01"[PDAT] : "2011/04/15"[PDAT]))

Morbidity and mortality

1. Sick*
2. "wounds and injuries"[MeSH Terms] OR "wounds and injuries"[All Fields] OR "injury"[All Fields])
3. "accidents"[MeSH Terms] OR "accidents"[All Fields] OR "accident"[All Fields]
4. "ill health"[All Fields] OR "illness"[All Fields]
5. Morbid*
6. Nutrition*
7. "nutritional support"[MeSH Terms] OR "nutritional support"[All Fields]
8. "nutritional requirements"[MeSH Terms] OR "nutritional requirement"[All Fields]
9. "malnutrition"[MeSH Terms] OR "malnutrition"[All Fields]
10. "undernutrition"[All Fields] OR "under-nutrition"[All Fields]
11. "undernourished"[All Fields]
12. "mortality"[Subheading] OR "mortality"[All Fields] OR "mortality"[MeSH Terms]
13. "death"[MeSH Terms] OR "death"[All Fields]
14. "tuberculosis"[MeSH Terms] OR "tuberculosis"[All Fields]
15. "malaria"[MeSH Terms] OR "malaria"[All Fields]
16. #68 OR #69 #OR #70 OR #71 OR #72 OR #73 OR #78 OR #79 OR #80 OR #81 OR #82
17. #83 AND ("humans"[MeSH Terms] AND English[lang] AND ("1990/01/01"[PDAT] : "2011/04/15"[PDAT]))

85 #17 AND (# 29 OR # 40 OR # 67 OR #84)

Total hits = 3,672

**Embase search**

1. economic AND 'growth'/exp OR economic AND 'development'/exp OR economic AND impact OR economic AND consequences OR economic AND 'analysis'/exp OR 'investments'/exp OR 'cost'/exp AND of AND 'illness'/exp OR 'disease'/exp AND burden OR 'cost'/exp AND minimization OR 'productivity'/exp OR wages OR salaries OR 'financing'/exp AND [1990-2011]/py
2. maternal OR maternity OR 'mother'/exp OR pregnant OR 'pregnancy'/exp AND disorder OR 'pregnancy'/exp AND outcomes OR 'pregnancy'/exp AND complications OR post AND partum OR postpartum OR post AND partal OR 'childbirth'/exp OR obstetric AND care OR breastfeeding OR 'breast feeding'/exp OR breastfeed OR 'lactation'/exp AND ([article]/lim OR [article in press]/lim OR [conference abstract]/lim OR [review]/lim) AND [humans]/lim AND [english]/lim AND [embase]/lim AND [1-6-1990]/sd NOT [15-4-2011]/sd AND [1990-2011]/py
3. 'child'/exp OR 'childhood'/exp OR 'paediatrics'/exp OR paediatric OR perinatal OR neonatal OR 'newborn'/exp OR 'infant'/exp OR 'baby'/exp OR 'toddler'/exp OR kid OR 'school'/exp AND 'age'/exp OR 'school age' OR 'juvenile'/exp OR underage OR 'under age' OR teen OR 'teenage'/exp OR 'adolescent'/exp OR 'birth'/exp AND 'weight'/exp OR low AND 'birth'/exp AND 'weight'/exp OR 'height'/exp AND [1990-2011]/py
4. reproductive OR 'reproduction'/exp OR 'contraception'/exp OR contraceptives AND agents OR 'contraceptive'/exp AND devices OR 'family'/exp AND 'planning'/exp OR 'family'/exp AND 'planning'/exp AND 'policy'/exp OR 'population'/exp AND 'control'/exp OR 'birth'/exp AND 'control'/exp OR 'abstinence'/exp OR 'abortion'/exp OR 'miscarriage'/exp OR 'stillbirth'/exp OR caesarean OR sexually AND transmitted AND diseases OR stds OR 'chlamydia'/exp OR genital AND 'herpes'/exp OR 'hepatitis'/exp OR 'gonorrhea'/exp OR 'syphilis'/exp OR genital AND 'warts'/exp OR 'hiv'/exp OR 'aids'/exp OR 'sexually' OR 'transmitted' AND 'infections' OR 'stis' OR 'std'/exp OR 'transmission'. AND [1990-2011]/py
5. sick OR 'sickness'/exp OR 'disability'/exp OR 'disable' OR 'prognosis'/exp OR 'injury'/exp OR 'accident'/exp OR 'ill health' OR 'illness'/exp OR 'morbidity'/exp OR 'nutrition'/exp OR 'nutritional' AND 'support' OR 'nutritional' AND 'requirement' OR 'malnutrition'/exp OR 'under' AND 'nutrition'/exp OR 'undernourished' OR 'nutritional' AND 'status' OR 'mortality'/exp OR 'death'/exp OR 'tuberculosis'/ exp OR 'malaria'/expAND [1990-2011]/py
6. #1 AND (#2 OR #3 OR #4 OR#5)
7. Total hits = 4,921

**CNIHAL and EconLit search (Embosco)**

1. TX Maternal OR maternity OR mother OR pregnant OR pregnancy OR pregnancy disorder OR pregnancy outcomes OR pregnancy complications OR post partum OR postpartum OR post partal OR childbirth OR obstetric care OR breastfeeding OR breast-feeding OR breastfeed OR lactation
2. TX Child OR childhood OR paediatrics OR paediatric OR perinatal OR neonatal OR newborn OR infant OR baby OR toddler OR kid OR school age OR school-age OR juvenile OR underage OR under-age OR teen OR teenage OR adolescent OR birth weight OR low birth weight OR height
3. TX Sick OR sickness OR disability OR disable OR prognosis OR injury OR accident OR ill-health OR illness OR morbidity OR nutrition OR nutritional support OR nutritional requirement OR malnutrition OR undernutrition OR undernourished OR nutritional status OR mortality OR death OR malaria OR tuberculosis
4. TX Reproductive OR reproduction OR contraception OR contraceptives OR contraceptive agents OR contraceptive devices OR family planning OR family planning policy OR population control OR birth control OR abstinence OR abortion OR miscarriage OR stillbirth OR caesarean OR sexually transmitted diseases OR STDs OR chlamydia OR genital herpes OR hepatitis OR gonorrhea OR syphilis OR genital warts OR HIV OR AIDS OR sexually OR transmitted infections OR STIs OR STD OR transmission
5. (#1 OR #2 OR #3 OR #4) AND limit to (Abstract Available; Published Date from: 19900101-20110531; English Language; Human; Publication Type: Abstract, Case Study, Clinical Innovations, Clinical Trial, Doctoral Dissertation, Drugs, Historical Material, Journal Article, Masters Thesis, Meta Analysis, Nursing Interventions, Research, Review, Statistics, Systematic Review; Language: English)
6. TX Economic growth OR economic development OR economic impact OR economic consequences OR economic analysis OR investments OR cost of illness OR disease burden OR cost minimization OR productivity OR wages OR salaries OR financing
7. #5 AND #6

Total hits = 2,339

**The National Bureau of Economic Research**

Under the working group paper:

- Children
- Health care
- Health economics

Total hit = 0

**POPLINE**

Under key words:

“ECONOMICS”; “ECONOMIC CONDITIONS”; “ECONOMIC DEVELOPMENT”; “ECONOMIC FACTOR”; “MACROECONOMIC FACTORS”

“CHILD HEALTH”; “CHILD HEALTH SERVICES”; “CHILD MORTALITY” ; “MATERNAL AND NEONATAL HEALTH”; “MATERNAL HEALTH”; “MATERNAL MORTALITY”; “MATERNAL NUTRITION” ; “MATERNAL HEALTH SERVICES”; “PREGNANCY”; “REPRODUCTION”; “REPRODUCTIVE HEALTH ISSUES”; “SEXTUALLY TRANSMITTED DISEASES”

Language restriction: only English articles

Total hits = 9,366
